# Supplementary material for: Atomic resolution electron microscopy in a magnetic field free environment
Source: Nat Commun. 2019 May 24;10:2308. doi: 10.1038/s41467-019-10281-2 (PMC6534592; doi:10.1038/s41467-019-10281-2)
Supplement: Supplementary file 1 — Supplementary Information [file 41467_2019_10281_MOESM1_ESM.pdf]

Supplementary Information

Atomic resolution electron microscopy in a magnetic field free environment

N. Shibata[1,2*], Y. Kohno[3], A. Nakamura[3], S. Morishita[3], T. Seki[1], A. Kumamoto[1], H. Sawada[3], T. Matsumoto[1], S.D. Findlay[4] and Y. Ikuhara[1,2]

[1]Institute of Engineering Innovation, The University of Tokyo, Bunkyo, Tokyo 113-8656, Japan.
[2]Nanostructures Research Laboratory, Japan Fine Ceramic Center, Atsuta, Nagoya 456-8587, Japan.
[3]JEOL Ltd., Akishima, Tokyo 196-8558, Japan.
[4]School of Physics and Astronomy, Monash University, Victoria 3800, Australia.

*To whom correspondence should be addressed: shibata@sigma.t.u-tokyo.ac.jp

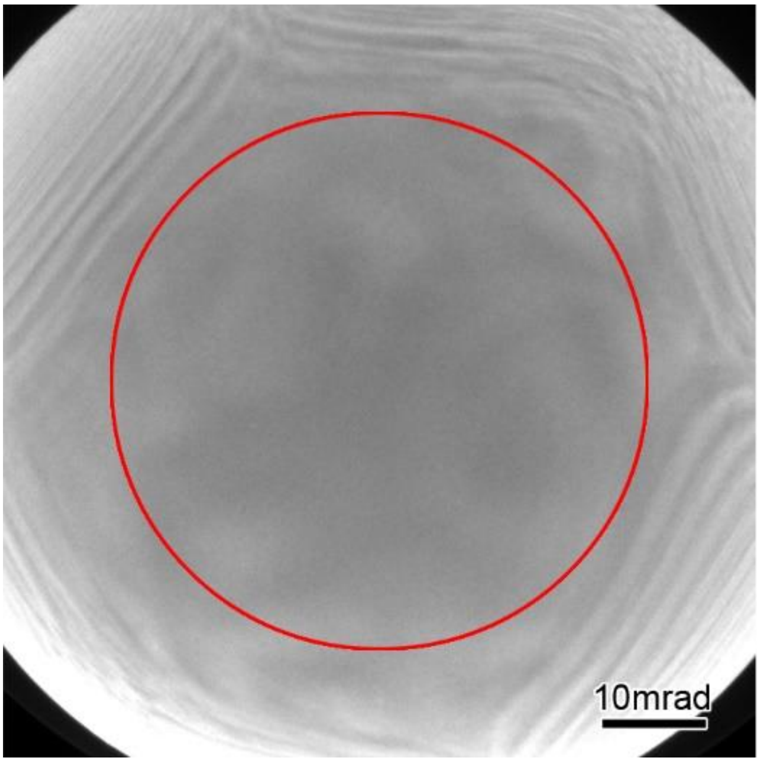

Supplementary Figure 1. Experimental Ronchigram obtained from an amorphous carbon film. It is clear that the flat phase region extends up to 26 mrad in semiangle (shown by the red circle).

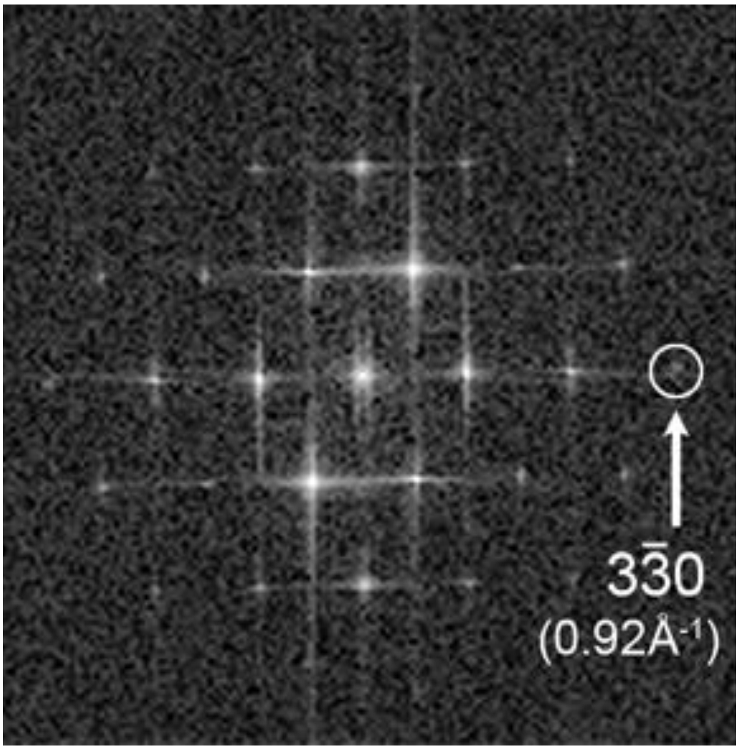

Supplementary Figure 2. The Fourier transform of the averaged GaN [111] ADF STEM image shown in Fig. 3**a**. It confirms information transfer to the 3$\bar{3}$0 (0.92Å) spacing.

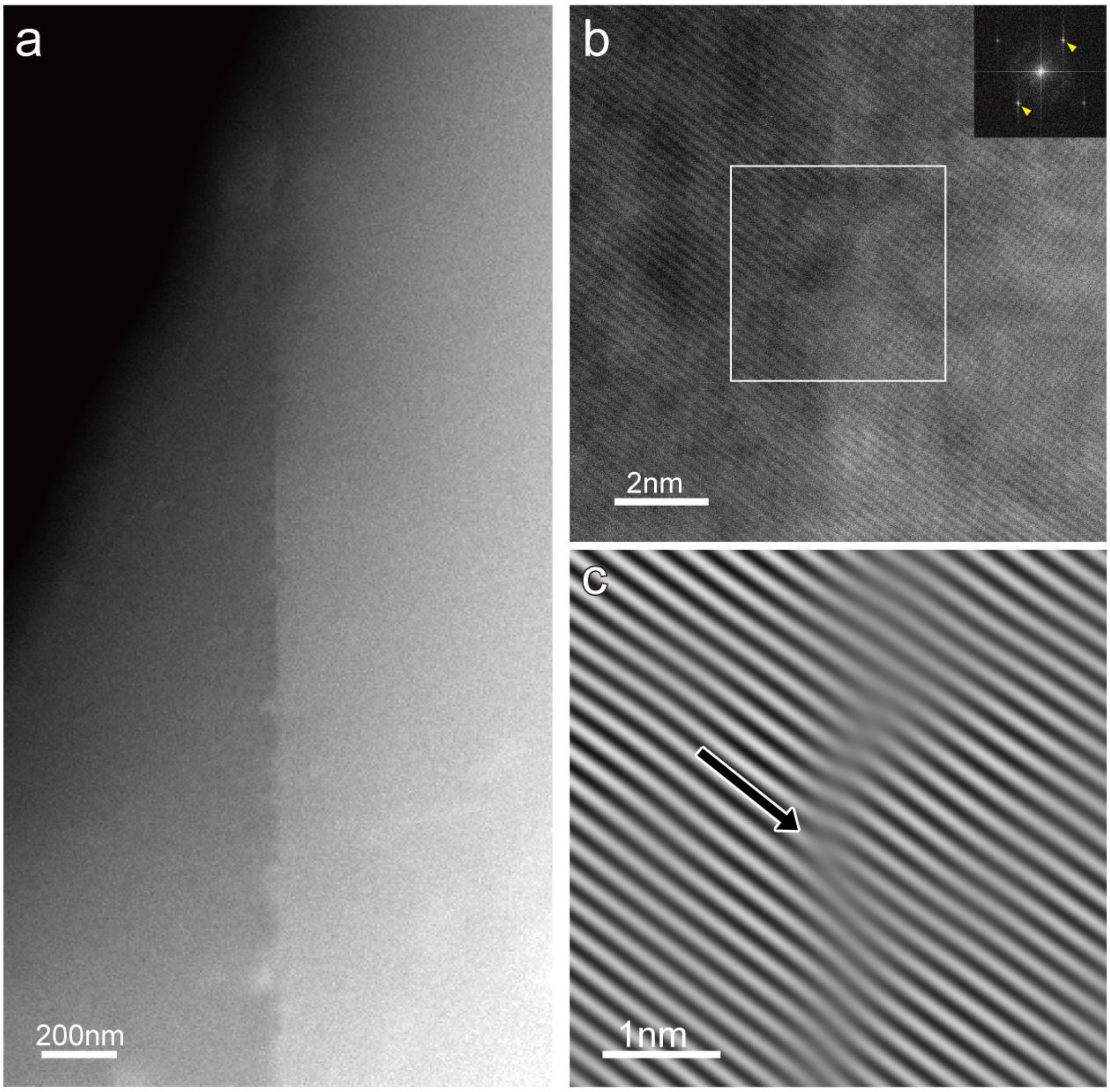

Supplementary Figure 3. ADF STEM images of a low-angle grain boundary in a grain-oriented silicon steel sheet (Fe-3%Si). (**a**) Low-magnification ADF STEM image showing the presence of a grain boundary. From the diffraction analysis, this grain boundary can be considered as a mix-type low-angle grain boundary. This low-angle grain boundary has both tilt and twist components, making it difficult to precisely align the crystal axes of both grains simultaneously. (**b**) Magnified image of the grain boundary. It is seen that the lattice is mostly continuous across the grain boundary. (**c**) FFT filtered image obtained from the white rectangular region shown in (**b**). As indicated by the arrow, there is a dislocation having an edge component. Thus, this grain boundary is composed of dislocations to accommodate orientation mismatch between the two grains.

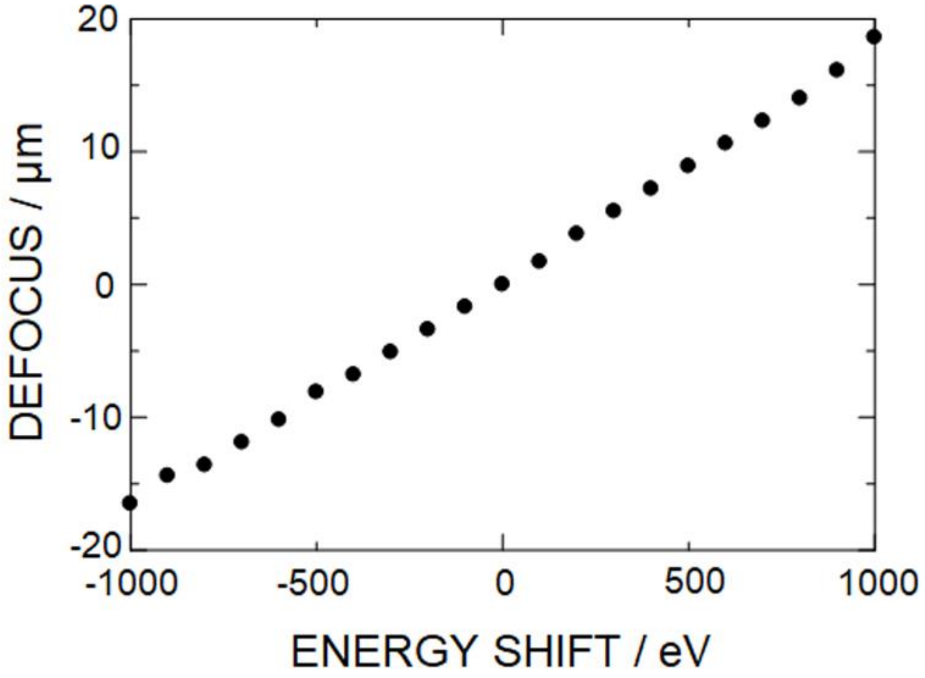

Supplementary Figure 4. The relationship between the defocus change and the energy shift of incident electron beam.

Year	Resolution (Å)	TEM or STEM	Aberration correction	Accelerating voltage (kV)	Manufacturers	Ref. no
2008	7.7	TEM	N/A	200	JEOL	1
2009	7	TEM	Cs correction	300	FEI	2
2012	5	TEM	Cs and Cc correction	300	FEI	3
2014	5	TEM	Cs correction	300	Hitachi	4
2015	5	STEM	Cs correction	200	JEOL	5
2015	2.4	TEM	Cs correction	1200	Hitachi	6
2017	6	TEM	Cs correction	300	FEI	7
Present results	0.92	STEM	Cs correction	200	JEOL	-

Supplementary Table 1. Previously reported attainable spatial resolution in magnetic field free transmission electron microscopy.

Lens type	Cs (mm)	Cc (mm)	focus length (mm)
The previous Lorentz-type objective lens system	95	18.6	17
The present objective lens system	16.9	3.45	3.14

Supplementary Table 2. Comparison between the previous JEOL Lorentz lens[1,8] and the present objective lens. It should be noted that this previous Lorentz type lens is not designed for ultra-high resolution imaging. It is clear that all the lens parameters are significantly improved in the present objective lens system, especially the much shorter focal length for large demagnification, which is essential to obtain a stable, noise-tolerant, atomic-size electron probe for STEM.

Supplementary References

1. Schofield, M.A., Beleggia, M., Zhu, Y. & Pozzi, G. Characterization of JEOL 2100F Lorentz-TEM for low-magnification electron holography and magnetic imaging. *Ultramicroscopy* **108**, 625-634 (2008).
2. Freitag, B. *et al.* Sub-nanometer resolution in field-free imaging using a Titan80-300 with Lorentz lens and image Cs-corrector at 300kV acceleration voltage. *Microsc. Microanal.*, **15** (Suppl. 2), 184-185 (2009).
3. Dunin-Borkowski, R.E. *et al*. Opportunities for chromatic aberration corrected high-resolution transmission electron microscopy, Lorentz microscopy and electron holography of magnetic minerals. *Microsc. Microanal.* **18** (Suppl. 2), 1708-1709 (2012).
4. Snoeck, E. *et al.* Off-axial aberration correction using a B-COR for Lorentz and HREM modes. *Microsc. Microanal.* **20** (Suppl. 3), 932-933 (2014).
5. McVitie, S. *et al.* Aberration corrected Lorentz scanning transmission electron microscopy. *Ultramicroscopy* **152**, 57-62 (2015).
6. Takahashi, Y. *et al.* Resolution assessment of an aberration corrected 1.2-MV field emission transmission electron microscope. *Microsc. Microanal.* **18** (Suppl. 3), 1865-1866 (2015).
7. Nagai, T., Kimoto, K., Inoke, K. & Takeguchi, M. Real-space observation of nanoscale magnetic phase separation in dysprosium by aberration-corrected Lorentz microscopy. *Phys. Rev. B.*, **96**, 100405(R) (2017).
8. Shindo D. *et al.* Electron holography of Nd–Fe–B nanocomposite magnets. *Scr. Mater.* **48** 851-856 (2003).
